# Supplementary material for: Evolution with a seed bank: The population genetic consequences of microbial dormancy
Source: Evol Appl. 2018 Jan 2;11(1):60–75. doi: 10.1111/eva.12557 (PMC5748526; doi:10.1111/eva.12557)
Supplement: Supplementary file 1 [file EVA-11-60-s001.docx]

**SUPPLEMENTARY MATERIAL**

**Simulations**

*Drift-mutation simulation* — The drift mutation simulation is an individual-based implementation of a Wright-Fisher model (Ewens, 2004). This simulation models a population of *N* active and *M* dormant individuals, where *c* randomly chosen individuals enter and exit the seed bank each generation. After entering and exiting the seed bank individuals within the active portion of the population acquire mutations, where each individual has a genome of fixed size (*G*) and average mutation rate $\mu$. The number of mutations are drawn from a Poisson distribution with mean *G**$\mu$. Mutations are randomly placed on the genome and all nucleotides have an equal probability of replacing the current nucleotide. The reproductive output of each haplotype is proportional to its frequency within the active portion of the population (i.e., the expected haplotype frequency in the next generation is equal to its current frequency), where the number of individuals of each haplotype in the next generation is chosen from a multivariate distribution. Evolutionary distance is estimated using the Jukes-Cantor model (Jukes & Cantor, 1969). The genetic diversity results from this simulation are presented in Fig. 3b and the substitution results are presented in Fig. 5b.

*Selection simulation* — To examine how that rate that individuals exit and enter a dormant state affects the trajectory of a beneficial mutation bound for fixation we simulated a Moran model with a seed bank component (Eriksson et al., 2008). The simulation tracks the trajectory of a newly arisen allele with selective advantage *s* = 0.10 (*B*) and of a neutral allele (*b*) in a population with *N* = 1,000 and *M* = 10,000 where c individuals exited the seed bank each generation. In a given generation there were *i* copies of *B* and *N-i* copies of *b* in *N* and *j* copies of *B* and *M-j* copies of *b* in *M*. One hundred values of *c* were chosen on a base 10 logarithmic scale, ranging in arithmetic values from 1 to 10,000. One thousand simulations were performed for each parameter combination. The results for this simulation are presented in Fig. 3a and Fig. 4b. The following equations represent the transition probabilities for the Moran model with a seed bank and selection.

$$p_{i\to i}=(\frac{i}{N}\cdot\frac{i}{N})(1-d)+\frac{i}{N}\cdot s(1-\frac{i}{N})(1-d)$$

$$p_{i\to j}=\frac{i}{N}\cdot d$$

$$p_{i\to(N-i)}=\frac{i}{N}(1-\frac{i}{N})(1-s)(1-d)$$

$$p_{(N-i)\to(N-i)}=(1-\frac{i}{N})(1-\frac{i}{N})(1-d)$$

$$p_{(N-i)\to i}=\frac{i}{N}(1-\frac{i}{N})(1-d)$$

$$p_{(N-i)\to(M-j)}=(1-\frac{i}{N})d$$

$$p_{j\to j}=\frac{j}{M}(1-r)$$

$$p_{j\to i}=\frac{j}{M}\cdot r$$

$$p_{(M-j)\to(M-j)}=(1-\frac{j}{M})(1-r)$$

$$p_{(M-j)\to(N-i)}=(1-\frac{j}{M})r$$

Where $K=\frac{N}{M},$ $d=\frac{c\cdot K}{N}$, and $r=\frac{c}{M}$. All other probabilities are zero.

**Computing code**

All simulations were written in Python v2.7.13. Computing code and simulated data are publically available on GitHub.

**Flow cytometry**

*Sample collection* — A single colony of *Janthinobacterium* sp. KBS0711 was grown overnight in 10 mL of PYE medium with glucose and casamino acids in a 50 mL Erlenmeyer flask in a 25°C shaker. One liter of the medium consists of 2 g bactopeptone, 1 g yeast extract, 0.30 g MgSO4 x 7 H2O, 2 g glucose, and 1 g casamino acids autoclaved in 1 L of water. After 12 hours, 100 μL was transferred into 10 mL of fresh media. For a given sampling point, aliquots of 100 μL were sampled and put into Eppendorf tubes with 900 μL of ePure water four times. One diluted media aliquot was not inoculated as a control and the remaining tubes were inoculated with either 1) 1 μL eBioscience™ Fixable Viability Dye eFluor™ 660, 2) 1 μL BacLight™ RedoxSensor™ Green Vitality Kit (RSG), or 3) 1 μL of eFluor and 1 μL RSG. RSG is an indicator of bacterial reductase activity, an enzyme that catalyzes reduction chemical reactions and can indicate electron transport chain function. eFluor 660 is a viability dye that labels cells with permeabilized membranes, allowing for cells that are likely dead to be removed. Treatments with just eFluor were inoculated and then left to incubate in a 25°C room in the dark for 40 minutes. Treatments with just RSG were left to incubate in a 25°C room in the dark for 30 minutes, inoculated with RSG, and then left to incubate for 10 minutes. Treatments with eFluor and RSG were inoculated with eFluor, left to incubate for 30 minutes, inoculated with RSG, and left to incubate for 10 more minutes. Inoculation was timed this way to allow for each sample to spend the same total length of time in the same environmental conditions. All samples were then inoculated with 13.5 μL of 37% formaldehyde and placed into a -80°C freezer.

*Data generation* — At a later date, frozen samples were taken out of the -80°C freezer, left to defrost, and 10 μL of each sample were transferred to a flow cytometer tube with 1 mL of ePure water two times. The second set of flow cytometry tubes were inoculated with 5 μL of 4',6-diamidino-2-phenylindole (DAPI) DNA fluorescent dye and left to incubate for 10 minutes. All flow samples were run on an LSRII flow cytometer at the Indiaia University Bloomington Flow Cytometry Core Facility.

*Analysis* — Samples not containing the DNA stain DAPI were used as a control to determine whether a data point was from a cell. The criteria for whether a data point was a cell was determined by setting a threshold two times the standard deviation plus the mean for the distribution of DAPI fluorescence. Data points above the threshold were classified as cells. The same process was used to remove dead cells using eFluor 660. The distribution of RSG (i.e., metabolic activity) was examined from the remainder of the points. All raw flow cytometry data was analyzed in Python using the following libraries: FlowCytometryTools, Pandas (McKinney, 2010), Matplotlib (Hunter, 2007), SciPy, and NumPy (van der Walt et al., 2011)

**REFERENCES**

Eriksson, A., Fernstrom, P., Mehlig, B., and Sagitov, S. (2008). An Accurate Model for

Genetic Hitchhiking. *Genetics* 178, 439–451.

Ewens, W. J. (2004). Mathematical Population Genetics, I. Theoretical introduction.

Interdisciplinary Applied Mathematics (Vol. 27). NY: Springer.

Hunter, J. H. (2007). Matplotlib: A 2D Graphics Environment. *Computing in Science &*

*Engineering*, 9, 90-95,

Jukes, T. H., and C. R. Cantor 1969. Evolution of protein molecules. *Mammalian Protein*

*Metabolism,* 21–123.

McKinney, W. (2010). Data Structures for Statistical Computing in Python. *Proceedings*

*of the 9th Python in Science Conference*, 51-56.

van der Walt, S., Colbert, S. C., & Varoquaux, G. (2011). The NumPy Array: A Structure

for Efficient Numerical Computation. *Computing in Science & Engineering*, 13, 22-30.
